# Supplementary material for: In Vitro Glioblastoma Model on a Plate for Localized Drug Release Study from a 3D-Printed Drug-Eluted Hydrogel Mesh
Source: Cells. 2024 Feb 19;13(4):363. doi: 10.3390/cells13040363 (PMC10887613; doi:10.3390/cells13040363)
Supplement: Supplementary file 1 [file cells-13-00363-s001.zip › cells-2740289-supplementary.pdf]

Supplementary Information

In vitro Glioblastoma Model on-a-plate for Localized Drug Release Study from a 3D-Printed Drug-eluted Hydrogel Mesh

*Behnad Chehri [1], Kaiwen Liu[1], Golnaz Vaseghi[1], Amir Seyfoori[1] *, Mohsen Akbari[1,2]**

[1]Laboratory for Innovations in Microengineering (LiME), Department of Mechanical Engineering, University of Victoria, Victoria, BC, V8P 5C2 Canada

[2]Terasaki Institute for Biomedical Innovation, Los Angeles CA 90064, United States of America

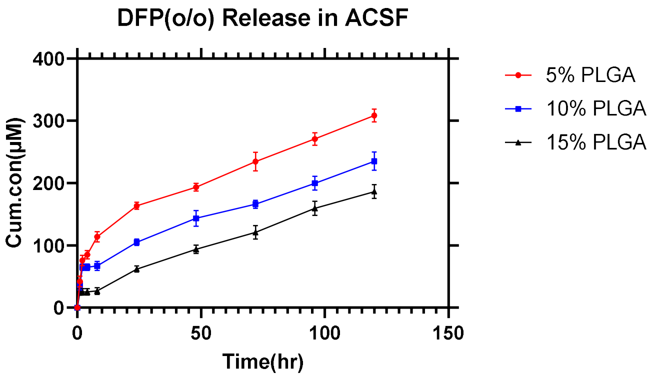

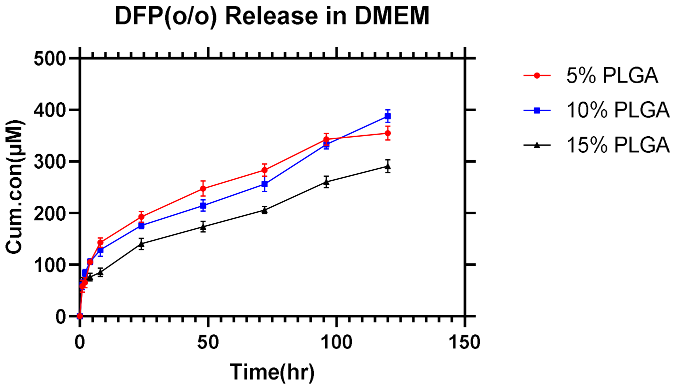

Fig S1. A) Overview of various release profiles of TMZ for various PLGA concentrations in ACSF, B) Overview of various release profiles of DFP for various PLGA concentrations in DMEM fabricated with the O/O method.

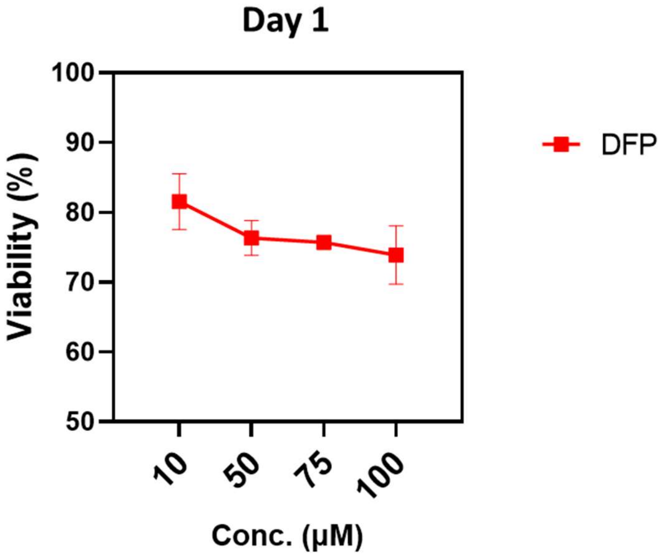

Fig S2. Cell viability of the U251 tumoroids after exposure to DFP for one day
